# Comparison between Micro-Computed Tomography and Cone-Beam Computed Tomography in the Assessment of Bone Quality and a Long-Term Volumetric Study of the Augmented Sinus Grafted with an Albumin Impregnated Allograft

**DOI:** 10.3390/jcm9020303

**Published:** 2020-01-21

**Authors:** Márton Kivovics, Bence Tamás Szabó, Orsolya Németh, Dóra Iványi, Bálint Trimmel, Ilona Szmirnova, Kaan Orhan, Eitan Mijiritsky, György Szabó, Csaba Dobó-Nagy

**Affiliations:** 1 Department of Community Dentistry, Semmelweis University, 1088 Budapest, Hungary; nemeth.orsolya@dent.semmelweis-univ.hu (O.N.); ivanyi.dora@dent.semmelweis-univ.hu (D.I.); 2 Department of Oral Diagnostics, Semmelweis University, 1088 Budapest, Hungary; drszbt@gmail.com (B.T.S.); trimmel.balint@dent.semmelweis-univ.hu (B.T.); dobo-nagy.csaba@dent.semmelweis-univ.hu (C.D.-N.); 3 Department of Department of Oro-Maxillofacial Surgery and Stomatology, Semmelweis University, 1085 Budapest, Hungary; szmirnova.ilona@dent.semmelweis-univ.hu (I.S.); szabo.gyorgy@dent.semmelweis-univ.hu (G.S.); 4 Dentomaxillofacial Radiology Department, Ankara University, Ankara 06560, Turkey; call53@yahoo.com; 5 Head and Neck Maxillofacial Surgery, Department of Otoryngology, Tel-Aviv Sourasky Medical Center, Sackler Faculty of Medicine, Tel-Aviv University, Tel Aviv 62431, Israel; mijiritsky@bezeqint.net

**Keywords:** sinus floor elevation, allograft, cone-beam computed tomography (CBCT), microcomputed tomography (micro-CT), bone quality, volumetric study, 3-year follow up

## Abstract

The purpose of our study was to compare micromorphometric data obtained by cone-beam computed-tomography (CBCT) and microcomputed-tomography (micro-CT) of the augmented sinus and to evaluate the long-term stability of the bone gain achieved using BoneAlbumin. Sinus lifts, and after 6-months, healing bone-biopsy and implant placement were carried out. Specimens were analyzed by micro-CT. A total of 16 samples were collected from nine patients (mean age 54.7 ± 6.5 years). Pre-, postoperative, and 3-year control CBCT-data were registered to determine from where the biopsy samples were harvested. Micromorphometric variables were calculated from the micro-CT- and CBCT-data, and their correlation was determined by Spearman’s test. The volume of augmented bone was calculated at the time of implant placement and after 3 years. A positive correlation was found between bone-volume fraction, trabecular-separation, open-, and total-porosity, while a negative correlation was found between trabecular-thickness obtained from CBCT- and micro-CT-data (*p* < 0.05). Mean volumetric reduction of 39.28% (11.88–60.02%) was observed. Correlation of CBCT- and micro-CT-data suggested that micromorphometric analysis of CBCT reconstructions of the augmented sinuses provided reliable information on the microarchitecture of augmented bone. CBCT as a modality might be adequate in the analysis of bone quality in the augmented sinus. At the 3-year, control sinus grafts showed volumetric stability.

## 1. Introduction

Axial, appendicular, and much of the craniofacial skeleton shows volume stability through adulthood. Loss of bone volume or mass usually occurs as a result of pathology in nutrition, hormonal maintenance, or tumors of bone tissue [1]. The alveolar ridge is a unique part of the skeleton in the sense that its formation is dependent on tooth eruption. Loss of teeth and the subsequent lack of functional loading of the alveolar ridge through periodontal ligaments leads to alveolar atrophy [2,3,4,5,6]. Sinus pneumatization is the expansion of the volume of the maxillary sinus at the expense of neighboring anatomical structures, such as the alveolar ridge [7,8]. Alveolar atrophy and sinus pneumatization combined may result in the decrease of bone volume in the edentulous posterior maxilla to the extent that precludes rehabilitation with dental implant borne restorations. Sinus floor elevation is a safe and predictable surgery to re-establish the bone volume required for implant placement [9].

Several bone-graft materials have been successfully applied in sinus floor elevation [10,11,12,13]. Allogenic bone overcomes donor site morbidity, and its available quantity is not restricted by patient-specific anatomical characteristics of the donor regions selected for bone harvesting. According to preclinical studies, albumin impregnation induces mesenchymal stem cell growth on the surface of freeze-dried bone allograft [14,15,16]. Studies show that allografts completely remodel into the newly formed bone of the maxilla when used as a graft material for sinus floor elevation [17,18].

Bone grafts in the maxillary sinus undergo initial resorption, a phenomenon known as re-pneumatization. However, the volume of the augmented bone stabilizes within 2-3 years after prosthetic loading of the implants as functional loading of the implant supporting bone counteracts the resorptive tendencies of the endosteum of the maxillary sinus [19,20,21].

There is no consensus in the literature regarding the accurate definition of bone quality of the implant recipient bone. However, the term bone quality includes the degree of mineralization, cortical bone thickness, and trabecular bone morphology [22]. Poor bone quality of dental implant recipient bone is associated with lower primary stability and higher implant failure rates. Primary implant stability affects whether the clinician opts for submerged or non-submerged implant placement protocols and determines the time of prosthetic loading. Therefore, assessment of bone quality prior to dental implant placement is essential [23,24].

Micro-computed tomography (micro-CT) is a time-efficient and reproducible method to study hard tissue specimens in high resolution. Studies show that morphological measurements by micro-CT reconstruction correlate highly with histomorphometric results, which is regarded as the gold standard for the evaluation of bone microarchitecture [25,26].

Compared to conventional computed tomography (CT), cone-beam computed tomography (CBCT) enables the imaging of the maxilla in high isotropic spatial resolution with low dose radiation [27]. Contrary to conventional CTs, quantitative grey value measurements in CBCT are unreliable and should be generally avoided [22]. CBCT-based bone density measurements are inherently inaccurate because of the configuration of the beam and flat-panel detectors, artifacts, and variations in the scanning conditions [28,29].

Numerous cadaver and clinical studies have been carried out to assess bone morphology of the native bone based on CBCT and micro-CT reconstruction [28,30,31,32,33,34,35,36,37,38,39]. However, evidence in the correlation of morphometric parameters between CBCT and micro-CT obtained data in augmented bone is lacking [37]. The main difficulty in the methodology of any clinical study correlating CBCT and micro-CT data is to determine the regions of interest (ROI) of the CBCT reconstruction from where the biopsy samples scanned by micro-CT are collected.

The first purpose of our clinical study was to compare micromorphometric data obtained by CBCT and micro-CT analysis of the augmented sinus and to determine whether CBCT as a modality was an adequate tool for the microarchitectural assessment of augmented bone. The second purpose of our study was to carry out a volumetric assessment of the re-pneumatization of the augmented sinuses to determine the long-term stability of the bone gain achieved by using the albumin impregnated allograft.

## 2. Materials and Methods

### 2.1. Study Design

The surgical interventions carried out in the study were thoroughly explained to the patients, and they signed informed consent forms. The study was approved by the Regional and Institutional Committee of Science and Research Ethics (52158-2/2015/EKU (0425/15)) and The Office of the Chief Medical Officer of The National Public Health and Medical Officer Service (IF-14561-10/2015). The study was conducted in accordance with the Helsinki declaration [40].

Periodontally healthy patients with an edentulous posterior maxilla who were more than 18 years of age were included in the present study. The exclusion criteria were as follows:

- History of systemic diseases that alter bone metabolism (osteoporosis, diabetes mellitus),
- History of medication known to alter bone remodeling (bisphosphonates, RANK ligand inhibitor monoclonal antibodies, corticosteroids),
- History of uncontrolled medical or psychiatric disorders,
- Inflammations of the paranasal sinuses or the alveolar process,
- History of tumors or irradiation therapy in the head and neck region,
- Unwillingness to return for follow-ups,
- Pregnancy,
- Smoking,
- Inability to perform proper oral hygiene.

### 2.2. Albumin Impregnated Allograft Preparation

Bone was harvested from cadavers or the femoral heads of live donors during primary hip replacement surgery under the operational license of the West-Hungarian Tissue Bank adhering to the guidelines set out in European Commission Directive 2004/23/EC. An autolyzed, antigen extracted allogeneic bone was produced following the Urist-protocol. Conservation was carried out by freeze-drying in aseptic conditions. After ethanol sterilization, bone grafts were submerged in sterile 10% human serum albumin solution for 60 seconds (low-salt-content Biotest human albumin infusion, Biotest Pharma GmbH, Dreieich, Germany). Preservation of the bone graft was finished with a second freeze-drying process [16].

### 2.3. Surgical Interventions

Patients were rinsed with 0.2% chlorhexidine gluconate solution for 1 min before surgery. Surgeries were carried out in local anesthesia. A mucoperiosteal trapezoidal flap was raised from a midcrestal incision and two releasing incisions. Osteotomies on the lateral wall of the maxillary sinuses were carried out with diamond burs. The membrane of the maxillary sinus was elevated carefully to avoid tear. Albumin impregnated freeze-dried bone allograft (BoneAlbumin, OrthoSera GmbH, Krems an der Donau, Austria) was packed in the base of the sinuses with light force. The lateral wall defect was covered with a two-layered resorbable porcine collagen membrane (Bio Gide, Geistlich GmbH, Wolhusen, Switzerland) and fixed by titanium pins (Titan Pin Set, Ustomed Instruments Ulrich Storz GmbH & Co. KG, Tuttlingen, Germany). The full-thickness flap was mobilized to allow tension-free primary closure. The wound was closed by single interrupted sutures. Antibiotics (amoxicillin-clavulanate 1 g, twice a day for 5 days or, in case of allergy, clindamycin 300 mg 4 times a day for 4 days) were prescribed. The patients took non-steroid anti-inflammatory drugs (NSAIDs) (diclofenac 50 mg, 3 times a day for 3 days). Patients were required to use 0.2% chlorhexidine-gluconate mouthwash twice a day from the first day after surgery for 3 weeks. Sutures removal took place after 10 days. During the 6-month healing period, as temporary prostheses, patients received fixed bridges with distal cantilevers so that the prostheses made no contact with the augmented area or did not receive temporary prostheses at all. Surgical re-entry took place after 6 months under local anesthesia. At the site of planned implant placement instead of step by step drilling, a trephine drill with an external diameter of 3.5 mm and an internal diameter of 2.5 mm (330 205 486 001 025 Hager & Meisinger GmbH, Neuss, Germany) was used to collect a bone core biopsy sample. After final drilling, implants of at least 4 mm in diameter were placed nonsubmerged. The bone core biopsy samples were placed in 4% formaldehyde solution in 0.1 M phosphate buffer saline (PBS), pH 7.3, stored at 4 °C. Final prosthetic restorations were fixed after 3 months [40].

Altogether, 16 biopsy samples were taken from the augmented sinuses of 9 patients (3 males, 6 females), mean age 54.7 ± 6.5 years. Long-term clinical and radiological follow up took place after 3 years. One of the 9 patients (one sinus grafted and one biopsy sample taken) failed to attend the 3-year follow up.

### 2.4. Micro-CT Scanning

The bone core biopsy samples were scanned at a resolution of 5.9 µm (70 kV, 124 µA) using a microcomputed tomography (micro-CT) scanner (Skyscan 1172, Bruker micro-CT, Kontich, Belgium). A 0.5 mm aluminum filter was used to reduce image noise. The average scan duration was 25 min. To minimize ring artifacts, the air calibration of the detector was carried out prior to each scan. After the acquisition, raw images were reconstructed by using NRecon software (v.1.67.91.46., Bruker micro-CT, Kontich, Belgium).

### 2.5. Micro-CT Imaging Analysis

**Figure 1:**
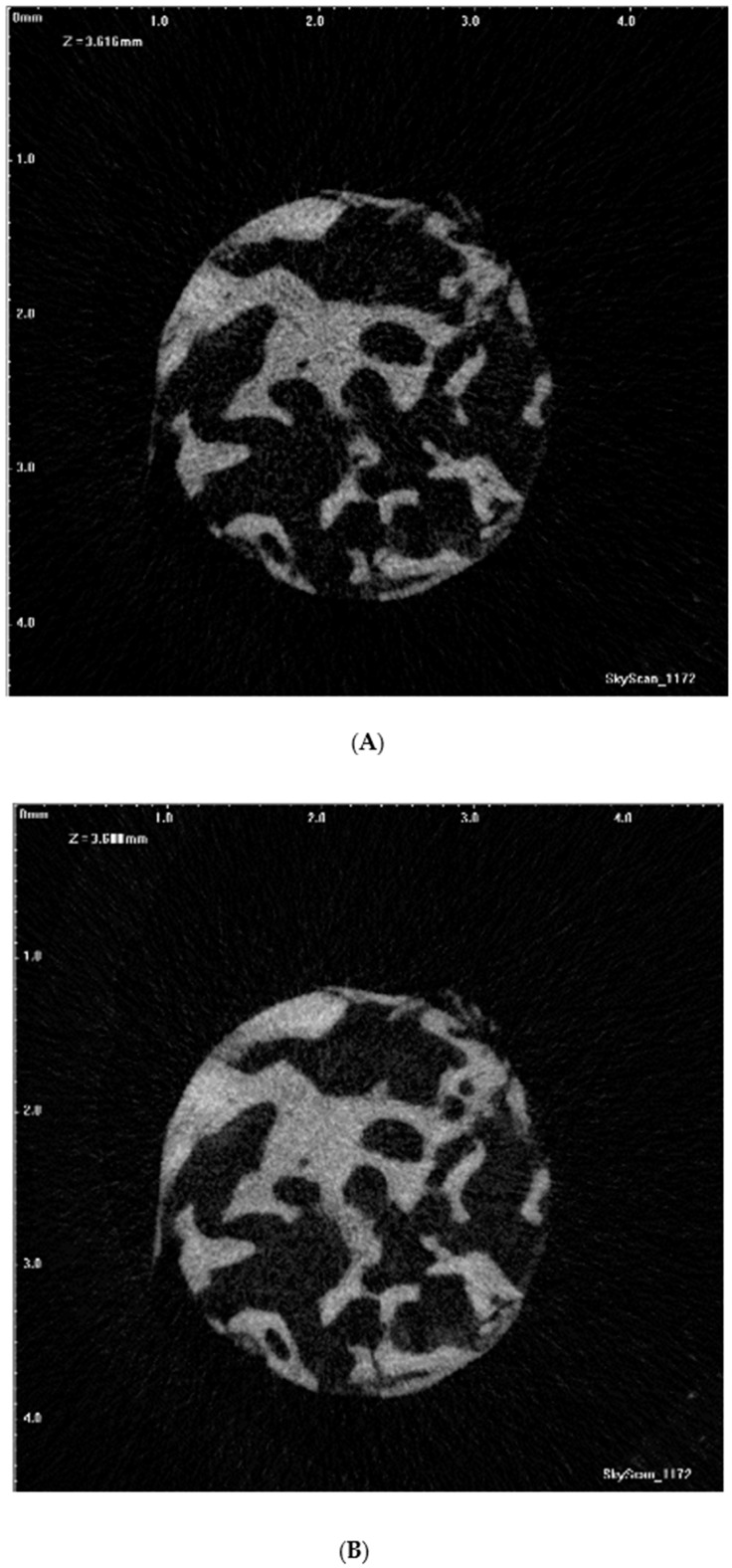
(**A**) An axial slice of reconstructed microcomputed tomography (micro-CT) dataset of the bone core biopsy specimen; (**B**) a maximum intensity projection (MIP) image of micro-CT axial slices.

Visualization and quantitative measurements of the samples were carried out using the NRecon software and CTAn (v.1.15.4.0, Bruker micro-CT, Kontich, Belgium), which applied the modified algorithm described by Feldkamp et al. to obtain axial, two-dimensional (2D) images [41]. For the reconstruction parameters, smoothing was fixed at zero; ring artifact correction and the beam artifact correction were set at 10% and 61%, respectively. The NRecon software was utilized to reconstruct the images obtained by the scanner to show 2D slices of the specimen. Figure 1A presents an axial slice of the reconstructed micro-CT image sequence of the bone core biopsy specimen. A maximum intensity projection (MIP) image was generated from micro-CT axial slices (Figure 1B), resulting in a possible comparable view of the core biopsy sample with the adequate axial slice of a postoperative reconstructed CBCT data.

The interpolated regions of interest (ROI) were selected on reconstructed 2D slices using CTAn software.

On the reconstructed images of the biopsy samples, the augmented area was selected as ROI for quantitative analysis [40,42].

In order to analyze the ROI of the whole specimen, the optimal grey value range was determined in the histogram. After the segmentation of the image, the software itself performed the analysis of micro morphometric parameters in an automatic way [43]. The morphometric variables relevant to our study calculated by CTAn software are listed in Table 1 [44,45]. 

### 2.6. CBCT Imaging

**Figure 2:**
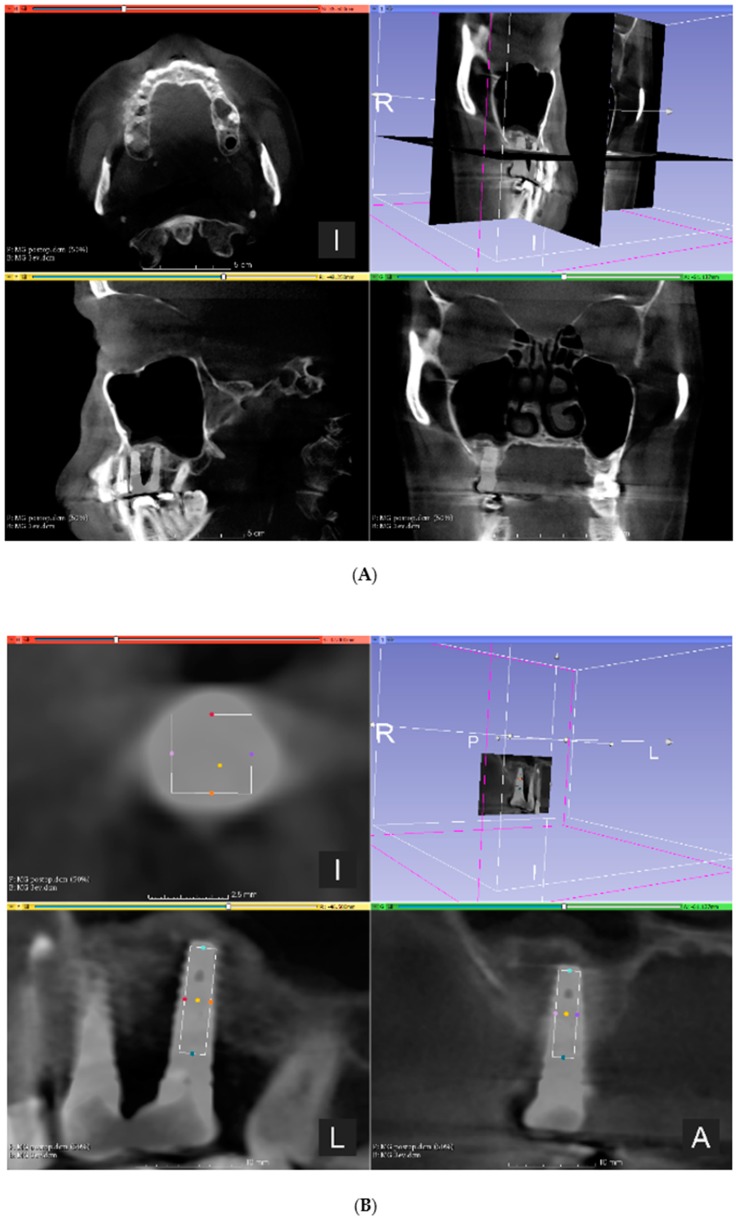
(**A**) The fused image of the postoperative and 3-year control cone-beam computed tomography (CBCT); (**B**) Identification of the region of interest (ROI) (position of the biopsy sample harvested during re-entry prior to dental implant placement) on the fused image is presented.

CBCT imaging (PaX-Reve3D, Vatech, Hwaseong, South-Korea) was carried out prior to sinus floor elevation to evaluate the anatomy and possible pathology of the maxillary sinuses (preoperative CBCT), then 6 months after bone augmentation prior to implant placement (postoperative CBCT), and 3 years after implant placement (3-year control CBCT). The scanning conditions were constant at 250 µm isotropic voxel size with 360° rotation, 89 kV tube voltage, 4.9 mA tube current, and 24 s exposure time for all specimens with a 15 × 15 cm field of view (FOV). To register preoperative, postoperative, and 3-year control CBCT data by anatomical landmarks, 3DSlicer 4.10.2 software (The Brigham and Women’s Hospital, Inc. Boston, MA, USA) was used. The registration of the image sequences was carried out manually by changing the opacity of the layers of the postoperative and 3-year control CBCT data of the same patient. The ROIs were identified by determining the central axis of the implants and placing a virtual cylinder on the postoperative CBCT image sequence with a diameter of 2.5 mm and the length of 8 mm on this axis corresponding with the inner dimensions of the trephine used for bone core biopsy harvesting. The fused image of the postoperative and 3-year control CBCTs and selection of the ROI on the fused volume is presented in Figure 2. The image sequences of selected ROIs were imported in CTAn software (v.1.17.7.2, Bruker micro-CT, Kontich, Belgium), and micromorphometric variables were calculated by the software.

**Figure 3:**
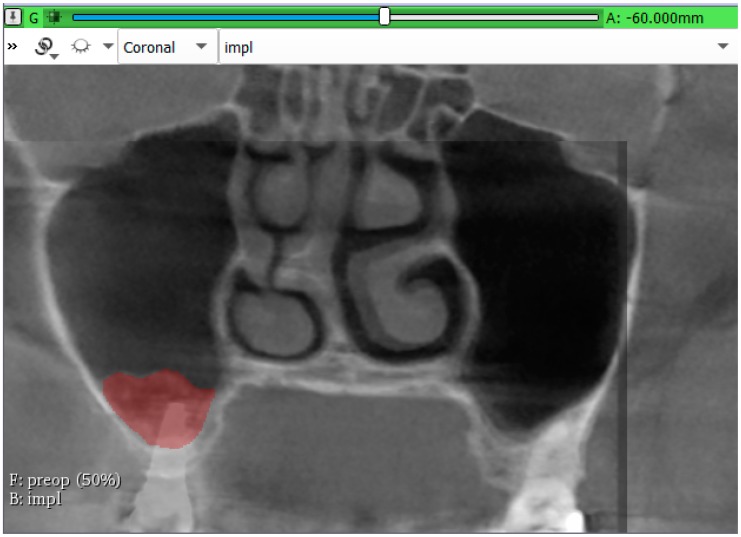
Volume measurement was carried out in the coronal section of fused images by changing the opacity of the layers of the pre- and postoperative CBCT, and—in this specific image—preoperative and 3-year control CBCT data of the same patient.

Volumetric measurements were carried out in the coronal sections of the CBCTs by marking the augmented area in each section using the segment editor module of the 3DSlicer 4.10.2 software (The Brigham and Women’s Hospital, Inc. Boston, USA). Augmented areas were determined by changing the opacity of the layers of the pre- and postoperative CBCT, and preoperative and 3-year control CBCT data of the same patient. The method of volume measurement is presented in Figure 3. The segment statistic module of the software was used to calculate the bone gain 6-month and 3-years after sinus elevations. Percental volumetric reduction was calculated in each sinus regarding the bone gain after 6-month healing as 100%.

### 2.7. Statistical Analysis

Correlation of the micromorphometric data obtained from the CBCT images and micro-CT images was determined by Spearman’s rank-order correlation. Statistical analysis was performed using the IBM SPSS Statistics 25 data analysis software program (IBM Corporation, New York, NY, USA). Values of *p* < 0.05 were considered statistically significant.

## 3. Results

### 3.1. Correlation of the Micromorphometric Data

Descriptive statistics of the micromorphometric data obtained from the micro-CT reconstructions of the bone core biopsy samples and their corresponding volume in postoperative CBCT images identified by implant positions on the 3-year control CBCTs are presented in Table 2.

A positive and statistically significant correlation was found between BV/TV, Tb.Sp, Po.V(op), Po(op), Po.V(tot), and Po(tot) micromorphometric parameters obtained from the CBCT and micro-CT data. A negative and statistically significant correlation was found between Tb.Th obtained from the CBCT and micro-CT data using Spearman’s rank-order correlation. P and R values of the correlation analysis are presented in Table 3.

### 3.2. Volumetric Assessment of the Augmented Sinuses

From the time of implant placement to the 3-year, control mean reduction of the augmented volume was 39.28% (11.88–60.02%). Descriptive statistics of the volumetric data and bone gain after 6-month and 3-year healing are presented in Table 4.

## 4. Discussion

CBCT is becoming the routine imaging method prior to dental implant placement due to its low dose radiation and high resolution compared to conventional CTs [27]. However, CBCT-based bone density measurements are controversial. CBCT data contains a larger amount of scattered X-rays because of the conical shape of the X-ray beam and the flat panel detectors. Heel effect is also coded in the conformation of the X-ray beam and detector: larger beamwidth results in a non-uniform angular distribution of beam intensity. The mean energy of the beam increases when it passes through solid objects known as the beam hardening effect [29]. These artifacts and scatter renders Hounsfield Unit (HU) measurements inapplicable in CBCT [22]. However, as CBCT is becoming a standard in the diagnostics of individual anatomy and pathology prior to bone augmentation and dental implant placement, determining bone quality from CBCT data is essential. Bone quality is a risk factor of implant survival, and its estimation prior to surgery helps the surgeon to determine the prognosis of survival of the implant and the possibility of immediate loading [24]. Literature suggests that, instead of HU, morphometric parameters obtained from CBCT data might be reliable predictors of bone quality when correlated to micro-CT data [36].

In an in vitro study, Hua et al. found that comparing the morphometric parameters of micro-CT and CBCT obtained data, bone mineral density (BMD) and fractal dimension (FD) correlated [31]. Ibrahim et al. compared CBCT and micro-CT datasets (Trabecular number (Tb.N), Tb.th, and Tb.Sp) in their cadaver study and found statistically significant and positive correlation; thus CBCT as a modality was adequate for the assessment of trabecular microarchitecture [32]. In their cadaver study, Kim et al. found that morphometric calculations from micro-CT and CBCT correlated positively except for Tb.Th [33]. Parsa et al. compared BV/TV data from CBCT and micro-CT reconstruction in their cadaver study and did not find a statistically significant correlation [35].

According to the results of Gonzales-Garcia et al., micro-CT obtained BV/TV data from bone core biopsy samples and CBCT obtained radiographic bone density (RBD) data of the sites of the biopsies showed a statistically significant positive correlation. Navigated biopsy taking and implant placement was carried out to determine the ROI of the CBCT [46]. In their clinical study, Monje et al. used a preoperatively fabricated acrylic resin with a metal rod at the future biopsy sites during CBCT to determine the ROI of the CBCT. They found that BV/TV, Tb.Th, and Tb.N correlated positively, while bone-specific surface (BS/BV), trabecular Tb.Sp, and trabecular pattern factor (Tb.Pf) correlated negatively with CBCT obtained RBD [34].

According to the literature, navigated implant placement is considered reasonably accurate (1–1.44 mm) with relatively high maximum deviations. The inner diameter of the trephines used in bone core biopsy (1.5–3.0 mm) is commensurable to the accuracy of navigated implant placement [47]. Therefore, in our study, the dental implant position on the control CBCT was used to accurately determine the ROI on the CBCT from where the bone core biopsy samples were taken. The 3DSlicer 4.10.2 (The Brigham and Women’s Hospital, Inc. Boston, USA) was used to merge the postoperative and 3-year control CBCTs and the site of the dental implants, and the inner diameter of the bone trephine revealed the position of the biopsy [48].

In our study, CBCT obtained BV/TV correlated positively and statistically significantly with micro-CT obtained BV/TV. This result corroborated conclusions from a previous cadaver and clinical studies [28,30,31,32,33,34,35,38,39]. 

Results of the present study regarding parameters of trabecular microarchitecture were controversial because Tb.Sp showed positive (R = 0.613) and Tb.Th, on the contrary, negative (R = −0.550) correlation between micro-CT and CBCT obtained data (*p* < 0.05). As a possible explanation, if the same object is scanned by a micro-CT and a CBCT device, the object is expected to be depicted in an extended volume on the CBCT dataset compared to the micro-CT image sequences, most probably as a consequence of the partial volume effect [49]. When a voxel contains tissues of different radiodensity, then the resulting CT value represents the average of their properties [29]. In our study, the CBCT voxel size was 250 µm, which is commensurable with the size of the trabecules. Hence, high-resolution CBCT with a voxel size of 100 µm or below might be beneficial for the assessment of trabecular bone microarchitecture prior to implant placement [49].

While BV/TV in the microarchitecture of trabecular bone represents the bone trabecules, porosity represents the bone marrow. The presence of closed pores is uncharacteristic of trabecular bone micromorphology and might be due to artifacts. The results of our study suggested that compared to open porosity, closed porosity is negligible. According to the results of the present study, CBCT and micro-CT obtained PoV(op), Po(op), PoV(tot), and Po(tot) data correlated positively and statistically significantly, which suggested that even at as low CBCT resolution as 250 µm, porosity measurement might be reliable in bone quality measurements.

In our study, BoneAlbumin, an albumin impregnated allograft, was used as filler in sinus floor elevations. To the best of our knowledge, our study was the first to evaluate the long-term results with this novel biomaterial in sinus grafting [44]. Volumetric results of our study showed that 3 years after dental implant placement, despite the re-pneumatization of the maxillary sinus, only one out of 15 dental implants protruded in the maxillary sinus. Clinical and radiological examination revealed no adverse reaction associated with the lack of bone, covering the apical portion of the implant. A total of 14 out of 15 implants were surrounded by augmented bone. No inflammatory signs were present in any of the treated sinuses.

## 5. Conclusions

In our study, a novel method was described to determine the regions of interest (ROI) of the CBCT reconstruction from where the biopsy samples scanned by micro-CT were collected by merging 3-year control and postoperative CBCTs.

Correlation of CBCT and micro-CT data suggested that micromorphometric analysis of CBCT reconstructions of the augmented sinuses provided reliable information on the microarchitecture of augmented bone area. CBCT as a modality might be adequate in the analysis of bone quality prior to implant placement in the augmented sinus.

Parameters of trabecular microarchitecture calculated from CBCT data at 250 µm might not be reliable in the microarchitectural assessment of augmented bone.

Porosity measurements from CBCT data might be reliable in bone quality measurements.

Application of the albumin impregnated allograft as a filler in sinus floor elevation provided long term volumetric stability of the augmented sinuses.

## Figures and Tables

**Table 1:** The micromorphometric variables calculated by the CTAn software (according to the manual Bruker microcomputed tomography (micro-CT) morphometric parameters measured by CT-analyzer software 1.15.4.0 by Bruker micro-CT) [44,45].

Abbreviation	Variable	Description	**Standard Unit**
BV/TV	Bone volume fraction	The relative volume of calcified tissue in the selected volume of interest (VOI).	%
Tb.Th	Trabecular thickness	Mean thickness of trabeculae, assessed using direct 3D methods.	µm
Tb.Sp	Trabecular separation	Mean distance between trabeculae, assessed using direct 3D methods.	µm
Po.V(op)	The volume of open pore space	The total volume of all open pores within the VOI is reported. An open pore is defined as any space located within a solid object or between solid objects, which has any connection in 3D to space outside the object or objects.	µm^3^
Po(op)	Open porosity (percent)	Percent of open porosity is the volume of open pores as a percent of the total VOI volume.	%
Po.V(tot)	The total volume of pore space	The total volume of all open and closed pores within the VOI is reported.	µm^3^
Po(top)	Total porosity (percent)	Percent of total porosity is the volume of total pores as a percent of the total VOI volume.	%

**Table 2:** Descriptive statistics of the micromorphometric data obtained from micro-CT reconstructions and of their corresponding volume in cone-beam computed tomography (CBCT) images.

		Unit	Mean	Minimum	Maximum
**BV/TV**	CBCT	%	81.289	37.872	98.423
	micro-CT	%	12.251	4.548	21.879
**Tb.Th**	CBCT	µm	1823.571	1122.201	2257.021
	micro-CT	µm	148.936	115.679	194.131
**Tb.Sp**	CBCT	µm	846.649	500.000	1767.296
	micro-CT	µm	875.978	332.867	1588.054
**PoV(op)**	CBCT	mm^3^	8.986	0.811	27.270
	micro-CT	mm^3^	45.826	10.322	85.207
**Po(op)**	CBCT	%	18.691	1.569	62.128
	micro-CT	%	87.744	78.112	95.451
**PoV(tot)**	CBCT	mm^3^	8.994	0.811	27.270
	micro-CT	mm^3^	45.828	10.324	85.207
**Po(tot)**	CBCT	%	18.711	1.577	62.128
	micro-CT	%	87.749	78.121	95.452

**Table 3:** R- and *p*-values of Spearman’s rank-order correlation between micromorphometric parameters obtained from the CBCT and micro-CT data.

Micromorphometric Parameter	R-Value	*p*-Value
BV/TV	0.550	0.034 *
Tb.Th	−0.550	0.034 *
Tb.Sp	0.613	0.015 *
PoV(op)	0.575	0.025 *
Po(op)	0.539	0.038 *
PoV(tot)	0.575	0.025 *
Po(tot)	0.550	0.034 *

* *p* < 0.05

**Table 4:** Volumetric changes of the augmented sinuses.

	Unit	Mean	Minimum	Maximum
Bone gain after 6-months	mm^3^	1623.32	815.79	2330.43
Bone gain after 3-years	mm^3^	981.19	371.89	1403.69
Volume reduction	%	39.28	11.88	61.02
